# Lipoprotein subfractions by nuclear magnetic resonance are associated with tumor characteristics in breast cancer

**DOI:** 10.1186/s12944-016-0225-4

**Published:** 2016-03-12

**Authors:** Vidar G. Flote, Riyas Vettukattil, Tone F. Bathen, Thore Egeland, Anne McTiernan, Hanne Frydenberg, Anders Husøy, Sissi E. Finstad, Jon Lømo, Øystein Garred, Ellen Schlichting, Erik A. Wist, Inger Thune

**Affiliations:** The Cancer Centre, Oslo University Hospital HF, N-0424 Oslo, Norway; Department of Circulation and Medical Imaging, Norwegian University of Science and Technology (NTNU), Trondheim, Norway; Department of Chemistry, Biotechnology and Food Science, Norwegian University of Life Sciences, N-1432 Aas, Norway; Public Health Sciences Division, Fred Hutchinson Cancer Research Center, Seattle, WA USA; Norwegian Directorate of Health, PO Box 7000, St. Olavs plass, N-0130 Oslo, Norway; Department of Pathology, Oslo University Hospital, N-0424 Oslo, Norway; Department of Breast and Endocrine Surgery, Oslo University Hospital, N-0424 Oslo, Norway; Department of Community Medicine, Faculty of Health Sciences, University of Tromsø, N-9037 Tromsø, Norway

**Keywords:** Lipoproteins, Subfractions, HDL, Breast cancer, Tumor, Progesterone receptor, Ki67

## Abstract

**Background:**

High-Density Lipoprotein (HDL)-cholesterol, has been associated with breast cancer development, but the association is under debate, and whether lipoprotein subfractions is associated with breast tumor characteristics remains unclear.

**Methods:**

Among 56 women with newly diagnosed invasive breast cancer stage I/II, aged 35–75 years, pre-surgery overnight fasting serum concentrations of lipids were assessed, and body mass index (BMI) was measured. All breast tumors were immunohistochemically examined in the surgical specimen. Serum metabolomics of lipoprotein subfractions and their contents of cholesterol, free cholesterol, phospholipids, apolipoprotein-A1 and apolipoprotein-A2, were assessed using nuclear magnetic resonance. Principal component analysis, partial least square analysis, and uni- and multivariable linear regression models were used to study whether lipoprotein subfractions were associated with breast cancer tumor characteristics.

**Results:**

The breast cancer patients had following means: age at diagnosis: 55.1 years; BMI: 25.1 kg/m^2^; total-Cholesterol: 5.74 mmol/L; HDL-Cholesterol: 1.78 mmol/L; Low-Density Lipoprotein (LDL)-Cholesterol: 3.45 mmol/L; triglycerides: 1.18 mmol/L. The mean tumor size was 16.4 mm, and the mean Ki67 hotspot index was 26.5 %. Most (93 %) of the patients had estrogen receptor (ER) positive tumors (≥1 % ER+), and 82 % had progesterone receptor (PgR) positive tumors (≥10 % PgR+). Several HDL subfraction contents were strongly associated with PgR expression: Apolipoprotein-A1 (β 0.46, CI 0.22–0.69, *p* < 0.001), HDL cholesterol (β 0.95, CI 0.51–1.39, *p* < 0.001), HDL free cholesterol (β 2.88, CI 1.28–4.48, *p* = 0.001), HDL phospholipids (β 0.70, CI 0.36–1.04, *p* < 0.001). Similar results were observed for the subfractions of HDL1-3. We observed inverse associations between HDL phospholipids and Ki67 (β -0.25, *p* = 0.008), and in particular between HDL1’s contents of cholesterol, phospholipids, apolipoprotein-A1, apolipoprotein-A2 and Ki67. No association was observed between lipoproteins and ER expression.

**Conclusion:**

Our findings hypothesize associations between different lipoprotein subfractions, and PgR expression, and Ki 67 % in breast tumors. These findings may have clinical implications, but require confirmation in larger studies.

**Electronic supplementary material:**

The online version of this article (doi:10.1186/s12944-016-0225-4) contains supplementary material, which is available to authorized users.

## Background

High-density lipoprotein (HDL), an important mediator of lipid homeostasis, transports and stores cholesterol for excretion [1], and cholesterol is a precursor of estrogen and progesterone [2], key factors in breast cancer development [3]. Moreover, increased levels of HDL cholesterol have been inversely associated with breast cancer development [4, 5], while increased levels of low density lipoprotein (LDL) cholesterol has been positively associated with breast tumor size, grade and proliferation [6]. HDL and apolipoprotein-A1 (Apo-A1) have also been shown to facilitate cholesterol efflux from white blood cells, thus decreasing the cellular lipid raft abundance [7, 8]. Whether HDL subfractions are associated with breast tumor characteristics is, however, less known.

Interestingly, low levels of HDL have been associated with increased levels of low-grade inflammation and proinflammatory cytokines [9–13], which in turn induce higher local estradiol levels and breast cell proliferation [14, 15]. We have recently observed that HDL-C levels, either alone or in combination with high levels of estrogen or progesterone, were associated with mammographic density phenotypes [16]. Moreover, lipid molecules have been shown to influence inflammation [9, 10], one of the hallmarks in cancer and breast cancer development [17] and prognosis [18], and elevated biomarkers of inflammation are associated with reduced survival among breast cancer patients. Notably, smaller and more dense HDL-particles may display different anti-inflammatory properties compared to larger HDL-particles [19], and may link lipoprotein subfractions to breast cancer development and breast tissue composition. Additionally, hypercholesterolemia, strongly associated with low HDL-C levels, may induce angiogenesis [20]. Thus, there is a biological plausibility for an association between lipoproteins, estrogen, progesterone and breast cancer development and prognosis [4, 6, 21]. To our knowledge, studies evaluating subfractions of lipoproteins have been limited to patients with cardiovascular disease, and have not yet included cancer patients [22–26].

The lipoprotein particle distributions have a high potential for improving the diagnostics of metabolic disorders [27], of potential importance for breast cancer development and treatment, and in particular among those with other comorbid conditions e.g., diabetes [28, 29]. Detecting metabolites downstream of gene- and protein activity, that influence endogenous metabolomic processes of potential importance for breast cancer development, has been enabled by emerging metabolomic profiling technologies. Magnetic resonance (MR) metabolomics has become one of the key methods in this research area [30]. Lipidomics refers to the use of analytical methods to identify and quantify lipid components in a biological matrix, such as biological fluids [31]. Recently very low density lipoprotein (VLDL) was associated with transport capacity of lipids to cancer cells [32]. Thus, to study lipidomics in more detail in relation to breast cancer development, we questioned whether a patient’s lipid profile, as visualized by the explorative lipoprotein subfraction method, may be associated with the histopathological characteristics of breast tumors [33, 34].

Thus, the main aim of this explorative, hypothesis generating study was to investigate the association between serum metabolomic lipoprotein subfractions and their contents of cholesterol, free cholesterol, phospholipids, apolipoprotein-A1 and apolipoprotein-A2, using nuclear magnetic resonance (NMR), and different breast tumor characteristics.

## Methods

### Participants and study design

A total of 60 breast cancer patients, aged 35–75 years, with newly diagnosed DCIS grade 3 and invasive breast cancer (histologically verified), stages I-II, were included in a clinical breast cancer study during 2011–2013 at the Cancer Center, Oslo University Hospital (OUS), St. Olavs Hospital, Trondheim, and Vestre Viken HF, Drammen. Women with known severe illnesses (i.e., heart disease, diabetes), were excluded. In the present study only women with histological verified invasive breast cancers were included: four women with DCIS grade 3 were excluded, thus 56 women diagnosed with invasive breast cancer were included in the present study.

### Assessment of clinical variables

Baseline patient characteristics, clinical data, and study measurements were assessed before treatment (surgery, radiation, chemotherapy) by trained study nurses and senior oncologists. Anthropometric measurements were performed with participants wearing light clothing and no footwear. Height was measured to the nearest 0.5 cm, and weight to the nearest 0.1 kg on an electronic scale, and BMI (kg/m^2^) was calculated. Blood pressure (BP) was measured three times (Dinamap-Pro Care 300), with the patient sitting in a resting position. The second measurements were used in the analysis.

Blood samples were drawn after overnight fasting. Total cholesterol, HDL-C, and triglycerides were measured in fresh sera at the Department of Clinical Chemistry, OUS, Ullevål (Roche Diagnostics/Cobas Integra 800- Cobas 8000, Mannheim, Germany, www.roche.com). Cholesterol was determined enzymatically using cholesterol esterase and cholesterol oxidase, intra-assay coefficient of variance (CV) was 6 % and inter assay CV was 3 %. HDL-C was quantified by a direct assay using polyethylene glycolmodified enzymes and dextran sulphate. HDL-C’s intra assay CV was 7 %, and inter assay CV was 4 %. Serum triglycerides were assayed by enzymatic hydrolysis with lipase, and had an intra-assay CV of 21 %, and inter-assay CV of 4 %. LDL-C was calculated using Friedewalds formula. Apolipoproteins A and B were measured using Cobas c501, (Roche diagnostics) and had intra-/inter-assay CVs of 7 %/4 % and 7 %/5 %, respectively.

### Tumor characteristics

All breast cancer surgical specimens were histologically and immunohistochemically examined. Tumors were classified according to invasive histological type (ductal, lobular, others), histological grade (1-3), and tumor diameter was measured both macro- and microscopically (mm). Lymph nodes were investigated to detect macro- or micro-metastasis, using sentinel lymph node (SN) biopsy technique for identifying axillary metastases.

**Fig. 1:**
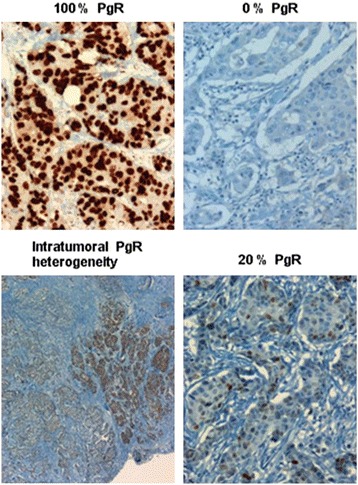
Different levels of Progesterone receptor status; 0 %, 20 %, 100 % and intratumoral heterogeneity

Tumors were routinely investigated with immunohistochemistry for selected markers: estrogen receptor (ER), progesterone receptor (PgR), human epidermal growth factor receptor 2 (HER2), and tumor cell proliferation (Ki67 hotspot index). The following antibodies were used: ER (clone SP1), PgR (clone 1E2), HER2 (Pathway anti-HER 2 kit, clone 4B5), and Ki67 (MIB1 antibody), all from Ventana, Roche Diagnostics (Oslo, Norway), except MIB1 which was provided by Dako (Oslo, Norway). Primary antibodies were visualized with Ultraview detection kit from Roche. ER, PgR and HER2 expression were measured according to the international guidelines (ASCO/College of American Pathologists [CAP]). Hormone receptor expression was given as the average percent of positive cells in the tumor. ER positive status was defined as ≥1 % ER-expressing tumor cells, and PgR positive status as ≥10 % PgR-expressing tumor cells. PgR expression may vary between different areas of the tumor as shown in Fig. 1 [35], and for 12 of the tumors the PgR positive fraction was given as above or below 50 %. These 12 tumors were set to PgR 50 % +. Immunohistochemic quantification of PgR is a reliable semiquantitative method used in clinical practice, but do have some limitations, but Immunohistochemic quantification of PgR has repeatedly, and recently been reported as a prognostic marker [36]. Tumors were investigated with HER2 Dual SISH in situ hybridization kit in order to determine HER2 status. The percentage of expression of Ki67 positive tumor cells was determined according to national and international guidelines [37, 38]. The Ki67 positive fraction was determined by counting at least 500 tumor cells in three representative high-power (x40 objective) fields in the most proliferative area of the tumor (“hot spot”), which was usually in the periphery. Ki67 score is defined as the percentage of positively stained cells undergoing active mitosis among the total number of malignant cells [37].

### Metabolic/lipidomic profiling- Magnetic resonance (MR) experiments

Venous fasting blood samples were collected in serum-tubes with no additives. The serum samples were stored at − 80 °C, until the time of metabolic profiling. The serum samples were slowly thawed at 4 °C. Aliquots of 150 μL were mixed with equal amounts of buffer solution and transferred to high-quality 3 mm MR tubes as described elsewhere [30].

The MR spectra were acquired using a Bruker Avance III 600 MHz/54 mm US-Plus (Bruker Biospin, Rheinstetten, Germany) operating at 600 MHz for proton (1H), equipped with a QCI cryoprobe. All spectra were recorded in an automatic fashion using a Bruker SampleJet and the ICON-NMR software (Bruker Biospin). Proton spectra were obtained at a constant temperature of 310 K (37 °C) using [1] a standard nuclear overhauser effect spectroscopy (NOESY) pulse sequence (Bruker: noesygppr1d) and [2] a Carr-Purcell-Meiboom-Gill (CPMG) pulse sequence with presaturation during the relaxation delay (Bruker: cpmgpr1d) to achieve water suppression, and to facilitate the detection of low-molecular-weight species by avoiding the large overlapped signals derived from large molecules, such as proteins and lipids. Measurement and processing were done in full automation using Bruker standard automation programs controlled by ICON-NMR (along with TopSpin). Chemical shift was calibrated to the middle of the alanine peaks at 1.50 ppm.

### MR spectra - MR based lipoprotein subclass analysis

Pre-processing of data was performed with MATLAB (Version 8.0.0.783 (R2012b); The Math Works, Natick, MA). The spectral region between 4.5 and 5.0 ppm was excluded to remove variation in water suppression efficiency. Spectra were normalized by setting the total spectral area to a constant value (=1) for all spectra to minimize possible differences in concentration between the samples.

**Fig. 2:**
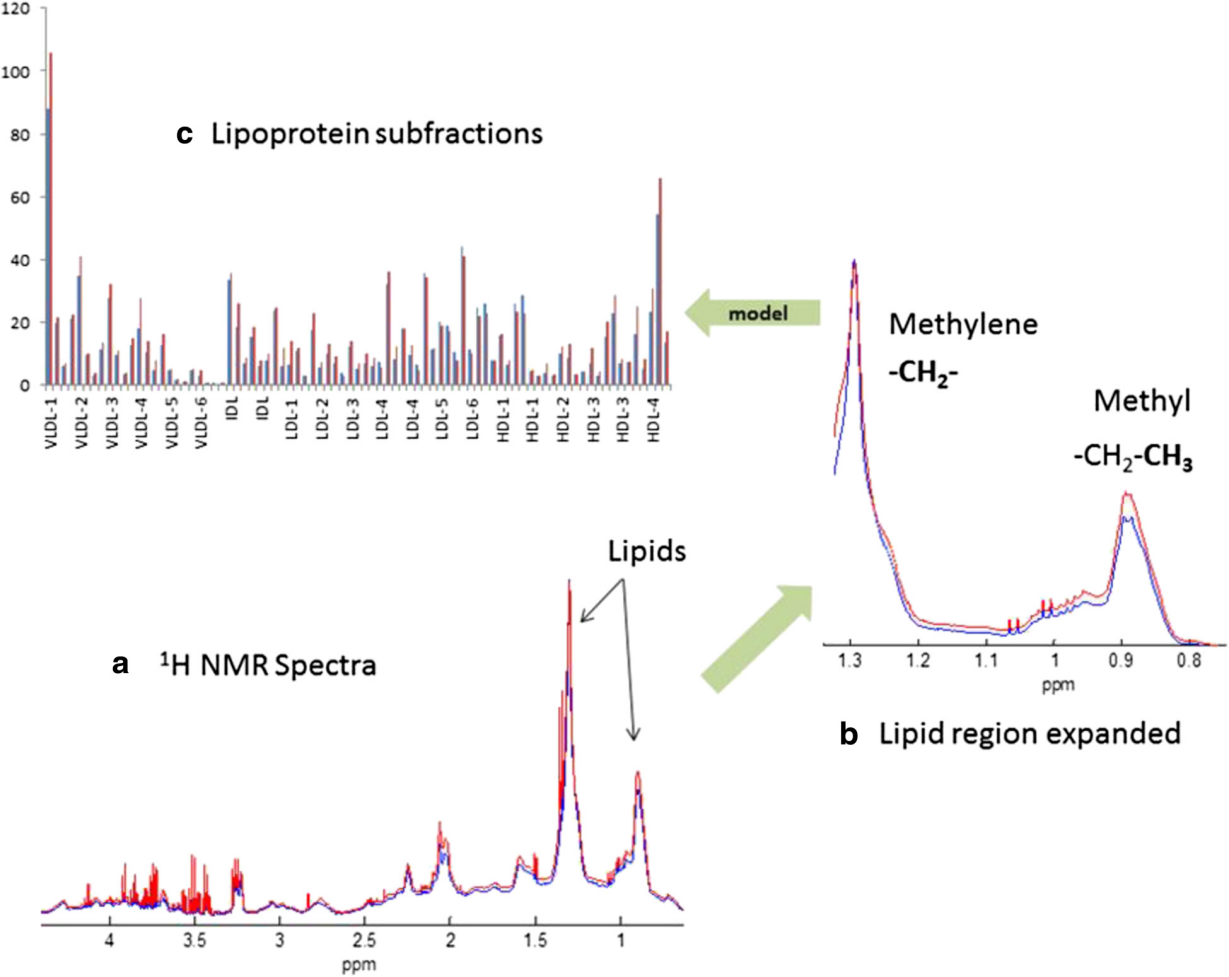
The relationship between serum NMR spectra and the lipoprotein subfractions. **a** Representative 1H NMR spectra from two individual breast cancer patients. **b** The two main lipid peaks represent methyl and methylene proton signals from lipid moieties within the lipoprotein particles. Minor differences in distribution of lipoprotein subfractions result in distinct line shape differences. **c** By regression-based modelling of the relationship between lipid signal line shape and lipoprotein subfractions, lipoprotein subfractions can be determined in new serum samples based on the 1H NMR spectrum

Calculation of lipoprotein related parameters from the plasma 1H NMR data was done at Bruker BioSpin GmbH Rheinstetten, Germany. For this, a regression model was applied which was developed by Bruker for NMR based lipoprotein subclass analysis [39], implementing a similar approach as established by Petersen et al. [40]. In brief, this approach is based on partial least squares modelling on a training data set which utilizes a combination of ultracentrifugation values on lipoprotein subclasses and 1H NMR spectra available for each plasma sample in a method training step. Model performance with respect to prediction quality and reliability is validated using cross-validation and test-set validation as employed e.g., in [41] and [27]. Once established, the resulting regression model can be used to predict lipoprotein related analytes directly from the 1H-NMR spectra of new plasma or serum samples not part of the training set, without further need for ultra-centrifugation. Using such a model, information extracted from the NMR data included the plasma content of very-low density lipoprotein (VLDL: <1.006 kg/l), intermediar low-density lipoprotein (ILDL: 1.006–1.019 kg/l), low-density lipoprotein (LDL: 1.019–1.063 kg/l), and high-density lipoprotein (HDL: 1.063–1.210 kg/l), as well as six subclasses of VLDL (VLDL-1, VLDL-2, VLDL-3, VLDL-4, VLDL-5, VLDL-6), six subclasses of LDL (LDL-1, LDL-2, LDL-3, LDL-4, LDL-5, LDL-6) and four subclasses of HDL (HDL-1, HDL-2, HDL-3, HDL-4). Subclasses were sorted according to increasing density and decreasing size in ascending order, respectively. Compositional information of main- and subclasses consists of the lipoprotein content concentrations of lipids, i.e., cholesterol, free cholesterol, phospholipids and triglycerides and apolipoproteins; Apo-A1, Apo-A2 and Apo-B. Model performance is comparable to the results reported in [27], as indicated by key model performance parameters summarized in Fig. 2 [39].

### Statistical methods

Descriptive statistics were used to describe the patient characteristics, including: age, anthropometric measurements, serum lipids (cholesterol, HDL-cholesterol, LDL-cholesterol, triglycerides, apolipoproteins) and tumor characteristics (tumor size, number of metastatic lymph nodes, estrogen and progesterone receptor, HER-2, and Ki-67 hot spot index). Continuous variables were assessed by means, standard deviations (SD), numbers and percentages, and the Chi-square test was used on categorical variables. Descriptive statistics of all lipoprotein subfractions were evaluated by means and SDs. Pearson’ correlations of the breast cancer tumor characteristic, and serum lipid variables were estimated and tested for significance. All lipoprotein variables were approximately normally distributed, hence no transformations were needed.

Based on plausible biological mechanisms hypothesized between lipid fractions and breast cancer development and prognosis, and previous works on MR metabolomics [42], we decided to use Principal Components Analysis (PCA) and Partial Least Square analysis (PLS) [42]. These statistical methods were chosen to investigate associations between lipoprotein subfractions and breast cancer tumor characteristics, while addressing the problem of multiple testing in our data with 56 samples and 105 different lipoprotein subfractions. The Principal Component Analysis (PCA) and Partial Least Square analysis (PLS) have proven powerful for dimension reduction and description of trends in large data sets. By using Principal Component Analysis (PCA), we ended up with eight components, and these eight components explained approximately 97 % of the variance of the 105 subfractions. In components with observed associations, we identified the specific lipoproteins with the highest scores. We then performed both uni- and multivariable linear regression between the high score lipoproteins and breast tumor characteristics. Potential confounding factors influencing tumor characteristics were tested in regression models, including: age (continuous), BMI (continuous), menopausal status (categorical), statin use (categorical), birth of children (categorical, yes/no), current smoking habits (categorical, yes/no), and previous oral contraceptive use (categorical, yes/no). Age, BMI and menopausal status were included as covariates in the final models.

To evaluate the results observed between lipoproteins and breast tumor characteristics using Principal Component Analysis (PCA), we also performed Partial Least Square analysis (PLS), for which only three components were needed. All *p*-values were two-tailed and considered significant if *p* < 0.05. The descriptive, correlation, principal component, and regression analyses were conducted with SPSS version 21.0 (IBM Corp. Armonk, NY, USA), and the partial least square analyses using the library of the R statistical package (http://cran.r-project.org/).

### Ethical considerations

All participants signed an informed consent form. The study was approved by the Norwegian Regional Committee for Medical Research Ethics.

## Results

The participating breast cancer patients had the following means: 55.1 years at diagnosis, BMI of 25.1 kg/m^2^, total cholesterol of 5.74 mmol/L, HDL-cholesterol of 1.78 mmol/L, LDL-cholesterol of 3.45 mmol/L, and Triglycerides of 1.18 mmol/L. The breast tumor size was on average 16.4 mm, and the mean Ki67 hotspot index was 26.5 %. 93 % of the breast tumors were ER+, 82 % were PgR+, and 7 % of the patients had hormone receptor negative disease (Table 1). BMI was not correlated with any of the tumor characteristics, but positively correlated with serum triglycerides, and inversely correlated with HDL-C (results not presented). The concentrations by means of the lipoprotein subfractions with lipid/lipoprotein contents are shown in Table 2.

**Table 1:** Descriptive statistics of the breast cancer patients by means and standard deviations (SD), *n* = 56

Characteristics	Mean (min.-max.)	SD
Age at diagnosis, years	55.1 (38–69)	7.89
Education, years	15.8 (8–24)	3.47
Postmenopausal, no (%)	38 (68 %)	
Systolic BP, mmHg	132 (87–184)	22.9
Diastolic BP, mmHg	77.4 (58–108)	11.9
Height, cm	167 (155–181)	5.97
Weight, kg	70.5 (49–97)	11.6
BMI, kg/m^2^	25.1 (20.0–33.2)	3.48
Statin use, no (%)	3 (5 %)	
Serum lipoproteins		
Cholesterol, mmol/L	5.74 (4.00–8.00)	1.00
HDL-cholesterol, mmol/L	1.78 (1.00–3.00)	0.49
LDL-cholesterol, mmol/L	3.45 (1.31–5.49)	0.98
Triglycerides, mmol/L	1.18 (0–3)	0.59
Apolipoprotein-A, mmol/L	1.67 (1–3)	0.29
Apolipoprotein-B, mmol/L	1.04 (0–2)	0.27
Tumor characteristics		
Tumor diameter, mm	16.4 (4–40)	8.52
Grade 1–3	1.96 (1–3)	0.71
Ki-67 hotspot, %	26.5 (1–81)	21.8
Nodal metastasis, no	0,75 (0–11)	2.13
ER positive, no (%)	52 (93 %)	
ER percent	88,1 (0–100)	26.9
PgR positive, no (%)	46 (82 %)	
PgR percent	64,2 (0–100)	36.8
Hormone receptor negative, no (%)	4 (7 %)	
HER2 positive, no (%)	3 (5 %)	

*Abbreviations*: *BP* blood pressure, *BMI* body mass index, *ER* estrogen receptor, *HER2* human epidermal growth factor 2, *HDL* high-density lipoprotein, *LDL* low-density lipoprotein, *PgR* progesterone receptor, *Ki 67* antigen Ki-67. ER positive when ≥ 1 %. PgR positive when ≥ 10 %

**Table 2:** The lipoprotein subfractions and their contents of lipids and lipoproteins in means and standard deviations (SD) by Nuclear Magnetic Resonance analyses

	Triglycerides	Cholesterol	Free cholesterol	Phospholipids	Apo-A1	Apo-A2	Apo-B
Total plasma, mg/dL	125 (57.2)	245 (58.9)	77.7 (17.8)	−	188 (36.7)	40.5 (9.53)	84.3 (23.1)
VLDL, mg/dL	70.1 (47.3)	16.4 (12.6)	8.81 (5.24)	18.9 (12.4)	−	−	5.29 (3.38)
VLDL1, mg/dL	40.5 (20.4)	5.85 (4.05)	2.33 (1.53)	7.25 (4.99)	−	−	−
VLDL2, mg/dL	11.6 (8.88)	2.94 (2.21)	0.96 (0.84)	3.77 (2.81)	−	−	−
VLDL3, mg/dL	7.80 (7.36)	2.23 (2.40)	1.19 (0.88)	4.20 (3.02)	−	−	−
VLDL4, mg/dL	6.14 (4.97)	3.01 (2.93)	1.34 (1.32)	4.18 (3.31)	−	−	−
VLDL5, mg/dL	1.86 (0.84)	1.12 (0.33)	0.46 (0.25)	1.70 (1.09)	−	−	−
VLDL6, mg/dL	3.65 (1.41)	0.15 (0.01)	0.02 (0.05)	0.40 (0.03)	−	−	−
ILDL, mg/dL	12.2 (7.23)	9.69 (5.65)	3.29 (1.64)	7.37 (3.44)	−	−	3.67 (1.52)
LDL, mg/dL	24.8 (5.99)	142 (45.5)	48.4 (13.6)	82.2 (23.1)	−	−	67.7 (19.8)
LDL1, mg/dL	7.32 (2.77)	24.2 (7.92)	8.80 (2.82)	9.66 (4.33)	−	−	9.74 (2.86)
LDL2, mg/dL	2.28 (0.77)	20.9 (8.44)	6.87 (2.94)	12.5 (4.71)	−	−	7.96 (3.08)
LDL3, mg/dL	2.43 (0.86)	25.4 (9.08)	10.1 (2.95)	14.4 (4.85)	−	−	10.6 (3.44)
LDL4, mg/dL	3.93 (1.24)	35.6 (11.6)	11.5 (3.14)	18.5 (6.43)	−	−	11.8 (3.16)
LDL5, mg/dL	2.68 (1.36)	26.0 (10.4)	9.65 (2.98)	14.5 (5.52)	−	−	12.2 (4.85)
LDL6, mg/dL	4.94 (1.59)	27.8 (12.1)	8.42 (3.34)	16.1 (6.20)	−	−	15.8 (6.49)
HDL, mg/dL	10.2 (4.60)	72.7 (19.4)	23.2 (5.56)	104 (25.3)	146 (32.5)	38.2 (8.74)	−
HDL1, mg/dL	4.39 (2.69)	21.3 (12.0)	9.22 (3.63)	28.3 (14.6)	27.3 (17.5)	3.36 (2.04)	−
HDL2, mg/dL	1.11 (0.88)	9.65 (3.42)	3.52 (1.23)	16.1 (5.78)	15.7 (6.76)	3.19 (1.68)	−
HDL3, mg/dL	1.96 (0.87)	13.8 (3.68)	4.63 (1.09)	22.5 (5.81)	31.1 (7.09)	7.52 (2.39)	−
HDL4, mg/dL	4.34 (1.17)	26.5 (5.16)	7.98 (1.51)	35.0 (5.57)	74.6 (10.8)	19.5 (4.72)	−

*Abbreviations*: *Apo* apolipoprotein, *HDL* high-density lipoprotein, *ILDL* intermediar low-density lipoprotein, *LDL* low-density lipoprotein, *VLDL* very low-density lipoprotein

We observed positive correlations between tumor characteristics and serum lipids. The continuous percentage PgR expression (Fig. 2) was inversely correlated with tumor grade and Ki67 hotspot index (Grade: correlation coefficient − 0.508, *p* < 0.001. Ki67: correlation coefficient − 0.577, *p* < 0.001) (Additional file 1: Table S1). Furthermore, we found positive correlations between PgR expression and both HDL and Apo-A (Additional file 1: Table S1).

**Fig. 3:**
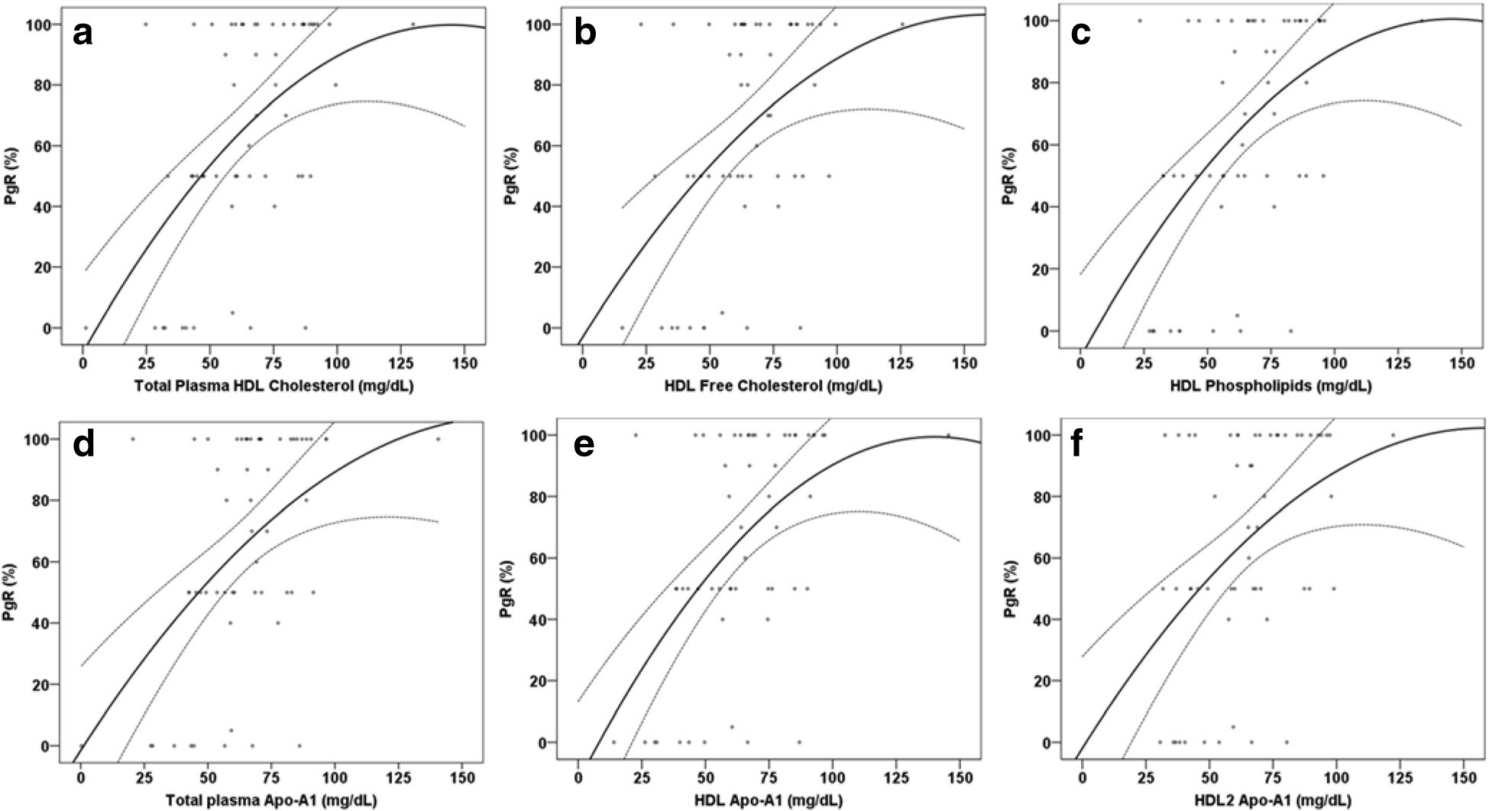
The multivariable linear association between lipoproteins and progesterone receptor status (%), adjusted for age, BMI and menopausal status with 95 % confidence interval. **a** HDL cholesterol, **b** HDL Free cholesterol, **c** HDL phospholipids, **d** total Apo-A1, **e** HDL Apo-A1, **f** HDL2 Apo-A1

By using Principal Component Analysis (PCA), in combination with uni- and multivariable linear regression analyses, total plasma apolipoprotein-A1 and the contents of cholesterol, free cholesterol, apolipoprotein-A1, apolipoprotein-A2, and phospholipids of HDL, HDL-1, HDL-2 and HDL-3, were associated with tumor PgR expression (Table 3). When performing multivariable linear regression, the following associations were found between PgR expression and lipids: total plasma Apo-A1 (β 0.46, *p* < 0.001), HDL-cholesterol (β 0.95, *p* < 0.001) (Table 3 and Fig. 3). We found no associations between the smaller and denser HDL-4 and PgR expression, and we found no association between any of the lipoproteins and ER status. In addition, we found inverse associations between HDL phospholipids and Ki67 (β -0.25, *p* = 0.008), and in particular with HDL1 and the contents of cholesterol, phospholipids, apolipoprotein-A1 and apolipoprotein-A2 and Ki67 (Table 3). The estimated β-coefficients (the linear gradient slope), and each unit increase of the lipoproteins were associated with a higher percentage level of PgR expression. These associations were similar in both the uni- and multivariable analyses (Table 3). The same analyses were also run by excluding the hormone negative cancers, with attenuated results (Additional file 2: Table S2).

**Table 3:** Principal Component analysis (PCA); the association between tumor characteristics and lipoprotein subfractions by NMR

	Univariable			Multivariable		
Tumor characteristics	β-coefficient	95 % CI	*p*-value	β-coefficient	95 % CI	*p*-value
Progesterone receptor (%)						
Total plasma Apolipoprotein A1, mg/dL	0.41	(0.17, 0.65)	0.001	0.46	(0.22, 0.69)	<0.001
HDL Cholesterol, mg/dL	0.86	(0.41, 1.31)	<0.001	0.95	(0.51, 1.39)	<0.001
HDL Free Cholesterol, mg/dL	2.64	(1.03, 4.26)	0.002	2.88	(1.28, 4.48)	0.001
HDL Phospholipids, mg/dL	0.66	(0.31, 1.01)	<0.001	0.70	(0.36, 1.04)	<0.001
HDL Apolipoprotein A1, mg/dL	0.50	(0.22, 0.78)	0.001	0.56	(0.29, 0.83)	<0.001
HDL Apolipoprotein A2, mg/dL	1.16	(0.06, 2.26)	0.040	1.53	(0.48, 2.58)	0.005
HDL1 Phospholipids, mg/dL	1.02	(0.39, 1.64)	0.002	0.96	(0.35, 1.57)	0.003
HDL1Cholesterol, mg/dL	1.20	(0.43, 1.97)	0.003	1.15	(0.41, 1.89)	0.003
HDL1Free Cholesterol, mg/dL	3.36	(0.80, 5.92)	0.011	3.44	(0.95, 5.93)	0.008
HDL1 Apolipoprotein A1, mg/dL	0.81	(0.29, 1.34)	0.003	0.76	(0.25, 1.27)	0.004
HDL1 Apolipoprotein A2, mg/dL	6.75	(2.20, 11.3)	0.004	6.39	(2.08, 10.7)	0.004
HDL2 Phospholipids, mg/dL	2.54	(0.95, 4.13)	0.002	2.30	(0.61, 3.98)	0.008
HDL2 Cholesterol, mg/dL	3.87	(1.19, 6.54)	0.005	4.42	(1.70, 7.15)	0.002
HDL2 Free Cholesterol, mg/dL	10.9	(3.48, 18.4)	0.005	11.6	(4.39, 18.9)	0.002
HDL2 Apolipoprotein A2, mg/dL	6.01	(0.26, 11.8)	0.041	6.74	(1.26, 12.2)	0.017
HDL2 Apolipoprotein A1, mg/dL	2.15	(0.79, 3.51)	0.003	2.29	(0.95, 3.63)	0.001
HDL3 Phospholipids, mg/dL	2.53	(0.95, 4.11)	0.002	2.88	(1.32, 4.43)	0.001
HDL3 Cholesterol, mg/dL	3.75	(1.28, 6.22)	0.004	4.44	(1.94, 6.95)	0.001
HDL3 Free Cholesterol, mg/dL	11.48	(3.06, 19.9)	0.008	13.5	(5.05, 22.0)	0.002
HDL3 Apolipoprotein A1, mg/dL	2.04	(0.73, 3.34)	0.003	2.38	(1.09, 3.66)	0.001
VLDL4 Cholesterol, mg/dL	−3.59	(−6.88,−0.31)	0.033	−3.76	(−7.16,−0.37)	0.031
VLDL 4 Free Cholesterol, mg/dL	−7.59	(−14.9, −0.27)	0.043	−7.75	(−15.3,−0.20)	0.044
Ki 67, %						
HDL Phospholipids, mg/dL	−0.25	(−0.47, −0.02)	0.033	−0.31	(−0.53,−0.08)	0.008
HDL1 Cholesterol, mg/dL	−0.48	(−0.96, −0.01)	0.048	−0.54	(−1.00,−0.07)	0.024
HDL1 Phospholipids, mg/dL	−0.40	(−0.80,−0.01)	0.043	−0.46	(−0.84,−0.08)	0.020
HDL1 Apolipoprotein A1, mg/dL	−0.31	(−0.64, 0.02)	0.066	−0.35	(−0.67,−0.03)	0.032
HDL1 Apolipoprotein A2, mg/dL	−3.23	(−6.01,−0.45)	0.024	−3.28	(−5.94,−0.62)	0.017
HDL2 Phospholipids, mg/dL	−1.08	(−2.06,−0.09)	0.033	−1.30	(−2.28,−0.31)	0.011
Nodal metastasis						
VLDL1 Triglycerides, mg/dL	0.03	(0.00, 0.06)	0.033	0.03	(0.00, 0.07)	0.036
VLDL1 Free Cholesterol, mg/dL	0.38	(0.01, 0.74)	0.045	0.41	(−0.01, 0.83)	0.057
LDL2 Free Cholesterol, mg/dL	−0.19	(−0.38,−0.00)	0.049	−0.17	(−0.37, 0.02)	0.084
LDL3 Free Cholesterol, mg/dL	−0.21	(−0.40,−0.03)	0.026	−0.20	(−0.40,−0.01)	0.049
Estrogen receptor (%)	No significant associations					
Grade 1–3						
Tumor size, mm						

Univariable and multivariable linear regression model. Multivariable model adjusted for age, BMI and menopausal status. 95 % Confidence Interval. Significance level *p* < 0.05

*Abbreviations*: *HDL* high-density lipoprotein, *VLDL* very-low-density lipoprotein, *LDL* low-density lipoprotein

We observed similar associations between lipoproteins and PgR expression in the Partial Least Square analysis (PLS) (Table 4); total plasma Apo-A1 (β 0.13, *p* = 0.002), HDL cholesterol (β 0.09, *p* < 0.001), but in addition it showed inverse trends between ILDL and PgR expression (*p* = 0.062), and between VLDL and PgR expression (*p* = 0.056), respectively (Table 4). Furthermore, we also observed an inverse association between total plasma triglycerides and tumor grade (Table 4). No association was found between the smaller and denser HDL4 and PgR, and no association was found between lipoproteins and ER expression in breast tumors.

**Table 4:** Partial Least Square analysis (PLS); the association between tumor characteristics and lipoprotein subfractions by NMR

Tumor Characteristic	β-coefficient	*p*-value
Progesterone receptor, %		
Total plasma Apolipoprotein A1, mg/dL	1.326 e-01	0.002
ILDL Free Cholesterol, mg/dL	−6.625 e-03	0.062
ILDL Phospholipids, mg/dL	−1.015 e-02	0.076
HDL Cholesterol, mg/dL	9.019 e-02	8.522 e-06
HDL Free Cholesterol, mg/dL	1.918 e-02	0.002
HDL Phospholipids, mg/dL	1.206 e-01	1.138 e-04
HDL Apolipoprotein A1, mg/dL	1.393 e-01	1.198 e-04
VLDL4 Cholesterol, mg/dL	−1.227 e-02	0.068
VLDL4 Free Cholesterol, mg/dL	−5.829 e-03	0.085
VLDL4 Phospholipids, mg/dL	−1.138 e-02	0.056
HDL1 Cholesterol, mg/dL	4.957 e-02	0.014
HDL1 Phospholipids, mg/dL	6.670 e-02	0.012
HDL1 Apolipoprotein A1, mg/dL	7.637 e-02	0.022
HDL1 Apolipoprotein A2, mg/dL	7.235 e-03	0.041
HDL2 Free Cholesterol, mg/dL	3.600 e-03	0.040
HDL2 Phospholipids, mg/dL	2.240 e-02	0.009
HDL2 Apolipoprotein A1, mg/dL	2.479 e-02	0.012
HDL3 Phospholipids, mg/dL	2.549 e-02	0.011
HDL3 Apolipoprotein A1, mg/dL	2.906 e-02	0.016
Tumor grade 1–3		
Total plasma Triglycerides, mg/dL	−2.694 e-03	0.011
HDL Apolipoprotein A1, mg/dL	−1.807 e-03	0.056
Ki67 %		
HDL Cholesterol, mg/dL	−3.252 e-02	0.060
HDL2 Cholesterol, mg/dL	−7.904 e-03	0.083
Estrogen receptor, %	No significant results	
Nodal metastasis, no		

*PLS* Partial Least Square (3 components included). Significance level *p* < 0.05

*Abbreviations*: *HDL* high-density lipoprotein, *ILDL* intermediar-low-density lipoprotein, *LDL* low-density lipoprotein, *VLDL* very-low-density lipoprotein

## Discussion

In the present explorative, and hypothesis generating study, we observed strong positive associations of metabolomic lipoprotein subfractions Apo-A1, HDL, and larger HDL subfractions’ contents of cholesterol, free cholesterol, phospholipids, and apolipoprotein-A1 with tumor PgR expression. No associations were observed between the smaller and denser HDL-4 and PgR expression. Furthermore, we observed an inverse association between the lipoprotein subfractions HDL1 and tumor cell proliferation, Ki67 index, and that very low-density lipoprotein (VLDL) was positively associated with nodal metastasis.

To our knowledge, this is the first study to investigate whether various lipoprotein subfractions are associated with breast cancer tumor characteristics. However, our results are supported by several observational studies linking cholesterol and lipoproteins to breast cancer development [4, 5] and survival [43]. Studies have also observed that there are distinct differences in the lipid metabolomics profiles comparing early and metastatic breast cancer [44]. The cholesterol metabolite, 27-OH cholesterol, has been observed to induce breast cancer cell proliferation and metastasis in hormone receptor positive cell lines, and 27-OH cholesterol is hypothesized to be one of the links between obesity and breast cancer [45, 46]. These findings support that also the type of cholesterol metabolite may play a role in breast cancer risk and prognosis.

The associations between lipoproteins and breast cancer development and prognosis may vary by type of lipoproteins, as we observed a positive association between the larger HDLs and PgR expression in breast tumors, but no associations between the smaller and more dense HDL4 and PgR expression. Recently, PgR expression < 20 % has been associated with poor prognosis [36, 47, 48], and patients initially diagnosed with PgR+ breast cancer had a worse outcome if recurrence of disease was PgR− [49]. Moreover, the TransAttack study showed that the lowest PgR percentage quartile had an unfavourable prognosis as compared to the highest quartile [50]. A proposed mechanism is that ligand activation of PgR induces PTEN expression and thereby inhibits the PI3K/AKT pathway [51]. In addition, PgR associates with ERα resulting in an increased anti-proliferative effect by a unique gene expression program that is associated with good clinical outcome. Moreover, copy number loss of the *PGR* gene is a common feature in ERα + breast cancers, and may explain lower PgR levels in a subset of cases [52]. These observations partly support our findings, and hypothesize that an association between the contents of lipoproteins and PgR expression and Ki67 may be useful in the identification of follow-up of high risk groups.

In the present study, we observed an inverse association between HDL1 and Ki67 hot spot index. Furthermore, we observed that very low-density lipoprotein (VLDL) was positively associated with nodal metastasis, and inversely associated with PgR expression. Thus, type of lipoprotein subfractions may be associated with several breast tumor characteristics, and not only PgR expression. These findings are partly supported by others, as high LDL levels were positively associated with breast tumor size, and Ki67 index, and also showed a trend towards more lymph node metastasis [6].

In an NMR study, high lipid spectra was associated with inflammation [53], supporting that metabolomic lipoprotein subfractions may play a role also in relation to inflammatory factors and pathways of importance for breast cancer development. Our observation that very low-density lipoprotein (VLDL) was positively associated with nodal metastasis, may be explained by the association between dyslipidaemia and vascular endothelial growth factor C (VEGF C) [54], as VEGF C promote nodal metastasis in combination with inflammatory cascades mediated particularly through tumor associated macrophages [55]. In addition, VLDL transports cholesterols, oxysterols and triglycerides from the liver to various tissues, and rapidly proliferating cancer cells require a constant supply of lipids for membrane biogenesis, protein modifications and steroid hormone production [32].

Studies suggest that the contents of lipoproteins may affect the development of several chronic diseases, and that the lipoprotein distribution may be of importance particularly in metabolic disorders [27]. A lipid reduced growth environment may attenuate cancer cell proliferation [32], and this knowledge may be helpful in designing new anti-tumor strategies [32]. Thus, it is important to further elucidate the different lipoproteins carrying various lipids, both in relation to size and density of the various lipoproteins. Larger HDL particles have been observed to reduce atherosclerotic development, and smaller HDL-particles are associated with obesity and metabolic syndrome [56]. These observations suggest shared biological mechanisms in the development of some chronic diseases. Recently, a difference between HDL-levels in breast cancer patients with, and without diabetes, was observed [29, 57]. HDL in diabetic breast cancer patients in contrast to nondiabetic patients may promote migration and invasion in both ER/PgR positive and PgR/ER receptor negative breast cancer through ERK and p38 MAPK pathways [29, 57]. Moreover, obese young breast cancer patients were found to have larger tumors, higher grade, and were more often ER negative and PgR negative [58], and the oxysterol, 27OH-Cholesterol, is associated with hormone receptor positive breast cancer cell proliferation [45]. These findings suggest that an association between type of lipoproteins and breast tumor characteristics may vary among breast cancer patients, depending on comorbidity (diabetes, obesity), age and menopausal status. In addition, previous studies have shown that the tumor expression of hydroxyl-methylglutaryl-coenzyme-A (HMG-CoA) reductase, the rate limiting enzyme in the cholesterol production, is associated with less aggressive tumor profiles, e.g., lower histological grade, estrogen and progesterone receptor positivity [28]. Previous studies have shown that increased lipid NMR signals have been attributed to inflammatory response in cancer [59].

Thus, several plausible biological mechanisms linking lipids and the contents of lipoproteins to breast cancer development support our findings. Low levels of HDL-C have been observed to stimulate inflammation through activation of the innate immunity [9, 11, 60], and to stimulate the production of neutrophils and proinflammatory macrophages inducing high levels of pro-inflammatory cytokines [10, 61, 62], higher local hormone levels and cellular proliferation in the breast [14, 63]. In addition, the HDL protein content [24, 26, 64] is also linked to inflammation. Of note, HDL and Apo-A1 may facilitate monocyte cholesterol efflux and thereby decrease cholesterol lipid rafts [8]. Moreover, an appropriate level of both HDL and Apo-A1 may down-regulate leukocyte activation [8]. Recently Apo-A1 was found to be down regulated in breast cancer patients [65]. In addition, oxidized LDL may trigger inflammation and PI3K, and reduce intracellular PTEN in human mammary epithelial cells [66].

All participating breast cancer patients were newly diagnosed with invasive breast cancer, and overnight fasting blood samples were drawn prior to surgery. The patients were informed about the breast cancer disease 1–5 days before blood sampling. Thus, any lifestyle changes including e.g., changes in dietary habits influencing their lipid profile, is less likely. Moreover, the participating women had an average BMI of 25.1 kg/m^2^, and BMI was inversely associated with HDL-C. Even though BMI was not associated with any of the tumor characteristics in this study, BMI has been shown to be a prognostic marker [67], and our final multivariable analysis included BMI as a covariate. The mean levels of cholesterol, and triglyceride observed in the present study are comparable with values observed among healthy women [68]. Blood lipid levels tend to remain stable, unless intensive intervention, such as lipid lowering medications, has occurred [69]. Importantly, lipid lowering medications were rarely used among our patients, and adjustments for lipid lowering medications did not influence our results. All clinical measurements were performed by trained personnel using validated methods at the research unit at the Oslo University hospital, Ullevål.

However, our study is explorative, and the study design was cross-sectional, and therefore we cannot establish cause-and-effect, and our results should thus be interpreted as explorative and hypothesis generating. Our sample size was small, and in combination with multiple testing, there is a risk of false positive results. In order to address these challenges, we used the robust statistical methods Partial Least Square analysis (PLS) and Principal Component Analysis (PCA) to support the results. The present method, developed and used to divide lipoproteins into 105 subfractions in breast cancer patients, is novel, and need to be validated in other studies, and importantly, later studies should compare results among breast cancer patients with healthy women. We also note that the expression of PgR in breast cancer tumors can vary across the area of the tumor [30], and the role of intratumoral heterogeneity of PgR expression may complicate any associations observed between lipoproteins and breast cancer prognosis. Thus, it would have been interesting to quantify the gene expression of *PgR* and look for associations with lipoproteins. However, in present day routine diagnostics, intratumoral heterogeneity of PgR expression is not reported, which contrasts with the present reporting of Ki67 in “hot spot” regions of the tumor.

Given the increase in obesity and unfavorable metabolic profiles worldwide, and the observed negative effect of obesity on breast cancer development and prognosis [67], there is a need for improved knowledge regarding the association between lipids and lipoproteins and breast cancer. Metabolomics, studies of metabolites in organic matrices, such as tissues and biofluids, as used in the present study, may detect new biological associations, as the organism’s metabolome may mirror disease impact [31]. This dynamic is promising in breast cancer research toward the discovery of new biomarkers of disease diagnosis, prognosis and treatment response [31]. In addition, robust multivariate statistical methods have been developed (PCA and PLS), and applied to handle large amounts of metabolomics data [42].

## Conclusions

In conclusion, we observed in this small explorative hypothesis generating study by using novel subfraction NMR methodology, that Apo-A1, HDL and HDL subfractions’ contents of cholesterol, free cholesterol, phospholipids, and Apo-A1 was associated with progesterone receptor expression. No association was observed between lipoproteins and ER expression, but we observed an inverse association between the lipoprotein subfractions HDL1 and Ki67 index, and very low-density lipoprotein (VLDL) was positively associated with nodal metastasis. Our findings suggesting that lipoprotein subfractions may be associated with breast tumor characteristics, of importance for tumor aggressiveness and prognosis, are supported by plausible biological mechanisms linking HDL and apolipoproteins to breast cancer development and prognosis. Our results are intriguing and encourage replications, but larger studies are needed, to define the clinical implications of these findings.

## Additional files

Additional file 1: Table S1. Correlations between breast cancer tumor characteristics and serum lipids. (DOCX 18 kb) Additional file 2: Table S2. Principal Component analysis (PCA); the association between tumor characteristics and lipoprotein subfractions by NMR among estrogen receptor positive (*n* = 52). (DOCX 16 kb)

## Abbreviations

Apo: Apolipoprotein
BMI: Body mass index
BP: Blood pressure
CI: Confidence interval
ER: Estrogen receptor
ERK: Extracellular signal-regulated kinases
HDL: High-density lipoprotein
HER2: Human epidermal growth factor 2
ILDL: Intermediar low-density lipoprotein
Ki 67: Antigen Ki-67
LDL: Low-density lipoprotein
MAPK: Mitogen activated protein kinase
NMR: Nuclear magnetic resonance
PCA: Principal component analysis
PgR: Progesterone receptor
PI3K: Phosphatidylinositol-3-kinases
PLS: Partial least square
PTEN: Phosphatase and tensin homolog
SD: Standard deviation
VLDL: Very low-density lipoprotein
