# Supplementary material for: Lipoprotein subfractions by nuclear magnetic resonance are associated with tumor characteristics in breast cancer
Source: Lipids Health Dis. 2016 Mar 12;15:56. doi: 10.1186/s12944-016-0225-4 (PMC4789271; doi:10.1186/s12944-016-0225-4)
Supplement: Additional file 1: Table S1. — Correlations between breast cancer tumor characteristics and serum lipids. (DOCX 18 kb) [file 12944_2016_225_MOESM1_ESM.docx]

| Supplementary table 1: Correlations between breast cancer tumor characteristics and serum lipids | | | | | | | | | | | | | |
| --- | --- | --- | --- | --- | --- | --- | --- | --- | --- | --- | --- | --- | --- |
|  |  | 1. | 2. | 3. | 4. | 5. | 6. | 7. | 8. | 9. | 10. | 11. | 12. |
|  |  |  |  |  |  |  |  |  |  |  |  |  |  |
| **1, Tumor diameter** | Correlation | | 0.029 | 0.213 | -0.033 | 0.292 | 0.145 | -0.159 | -0.164 | -0.112 | 0.024 | -0.168 | -0.179 |
|  | *p-value* | | *0.83* | *0.116* | *0.808* | *0.029* | *0.285* | *0.243* | *0.228* | *0.415* | *0.859* | *0.216* | *0.187* |
| **2, Grade** | Correlation | | 1 | 0.042 | 0.725 | -0.373 | -0.508 | -0.194 | -0.172 | -0.036 | -0.278 | -0.253 | -0.141 |
|  | *p-value* | |  | *0.759* | *<0.001* | *0.005* | *<0.001* | *0.152* | *0.206* | *0.796* | *0.04* | *0.059* | *0.299* |
| **3, Nodal metastasis** | Correlation | |  | 1 | 0.03 | -0.042 | -0.068 | -0.166 | -0.186 | -0.153 | 0.256 | -0.141 | -0.103 |
|  | *p-value* | |  |  | *0.827* | *0.76* | *0.62* | *0.222* | *0.169* | *0.264* | *0.059* | *0.3* | *0.451* |
| **4, Ki67 hot spot %** | Correlation | |  |  | 1 | -0.558 | -0.577 | -0.062 | -0.181 | 0.029 | -0.057 | -0.19 | 0.016 |
|  | *p-value* | |  |  |  | *<0.001* | *<0.001* | *0.648* | *0.182* | *0.831* | *0.679* | *0.161* | *0.91* |
| **5, ER %** | Correlation | |  |  |  | 1 | 0.463 | -0.052 | 0.078 | -0.067 | -0.066 | 0.129 | -0.148 |
|  | *p-value* | |  |  |  |  | *<0.001* | *0.703* | *0.567* | *0.628* | *0.634* | *0.345* | *0.278* |
| **6, PgR %** | Correlation | |  |  |  |  | 1 | -0.022 | 0.371 | -0.177 | -0.149 | 0.355 | -0.154 |
|  | *p-value* | |  |  |  |  |  | *0.874* | *0.005* | *0.197* | *0.276* | *0.007* | *0.258* |
| **7, Cholesterol** | Correlation | |  |  |  |  |  | 1 | 0.08 | 0.910 | 0.244 | 0.162 | 0.863 |
|  | *p-value* | |  |  |  |  |  |  | *0.558* | *<0.001* | *0.072* | *0.234* | *<0.001* |
| **8, HDL-chol** | Correlation | |  |  |  |  |  |  | 1 | -0.279 | -0.533 | 0.919 | -0.297 |
|  | *p-value* | |  |  |  |  |  |  |  | *0.039* | *<0.001* | *<0.001* | *0.026* |
| **9, LDL-chol** | Correlation | |  |  |  |  |  |  |  | 1 | 0.244 | -0.232 | 0.915 |
|  | *p-value* | |  |  |  |  |  |  |  |  | *0.073* | *0.088* | *<0.001* |
| **10, Triglycerides** | Correlation | |  |  |  |  |  |  |  |  | 1 | -0.295 | 0.419 |
|  | *p-value* | |  |  |  |  |  |  |  |  |  | *0.029* | *0.001* |
| **11, Apo-A** | Correlation | |  |  |  |  |  |  |  |  |  | 1 | -0.197 |
|  | *p-value* | |  |  |  |  |  |  |  |  |  |  | *0.145* |
| **12, Apo-B** |  | |  |  |  |  |  |  |  |  |  |  | 1 |
| Pearsons correlations, significance level p < 0.05.  Abbreviations: Apo, apolipoprotein; HDL, high-density lipoprotein cholesterol; LDL, low-density lipoprotein; ER, estrogen receptor; PgR, progesterone receptor; HER2, human epidermal growth factor 2; Ki 67, antigen Ki-67 | | | | | | | | | | | | | |
