# Supplementary material for: Lipoprotein subfractions by nuclear magnetic resonance are associated with tumor characteristics in breast cancer
Source: Lipids Health Dis. 2016 Mar 12;15:56. doi: 10.1186/s12944-016-0225-4 (PMC4789271; doi:10.1186/s12944-016-0225-4)
Supplement: Additional file 2: Table S2. — Principal Component analysis (PCA); the association between tumor characteristics and lipoprotein subfractions by NMR among estrogen receptor positive (n = 52). (DOCX 16 kb) [file 12944_2016_225_MOESM2_ESM.docx]

| Supplementary Table 2: Principal Component analysis (PCA); the association between tumor characteristics and lipoprotein subfractions by NMR among estrogen receptor positive (n=52) | | | | | | |
| --- | --- | --- | --- | --- | --- | --- |
|  | **Univariable** | | | **Multivariable** | | |
| **Tumor characteristics** | **β-coefficient** | **95% CI** | ***p*-value** | **β-coefficient** | **95% CI** | ***p*-value** |
| **Progesterone receptor (%)** |  |  |  |  |  |  |
| Total plasma Apolipoprotein A1, mg/dL | 0.38 | (0.15, 0.61) | *0.002* | 0.43 | (0.20, 0.65) | *<0.001* |
| HDL Cholesterol, mg/dL | 0.77 | (0.34, 1.21) | *0.001* | 0.86 | (0.43, 1.29) | *<0.001* |
| HDL Free Cholesterol, mg/dL | 2.39 | (0.85, 3.93) | *0.003* | 2.58 | (1.03, 4.12) | *0.002* |
| HDL Phospholipids, mg/dL | 0.58 | (0.24, 0.91) | *0.001* | 0.63 | (0.30, 0.97) | *<0.001* |
| HDL Apolipoprotein A1, mg/dL | 0.45 | (0.19, 0.70) | *0.001* | 0.51 | (0.25, 0.77) | *<0.001* |
| HDL Apolipoprotein A2, mg/dL | 1.00 | (-0.02, 2.03) | *0.054* | 1.37 | (0.36, 2.38) | *0.009* |
| HDL1 Phospholipids, mg/dL | 0.93 | (0.36, 1.50) | *0.002* | 0.91 | (0.33, 1.48) | *0.003* |
| HDL1Cholesterol, mg/dL | 1.09 | (0.39, 1.80) | *0.003* | 1.07 | (0.38, 1.77) | *0.003* |
| HDL1Free Cholesterol, mg/dL | 3.18 | (0.79, 5.56) | *0.010* | 3.16 | (0.80, 5.52) | *0.010* |
| HDL1 Apolipoprotein A1, mg/dL | 0.77 | (0.29, 1.25) | *0.002* | 0.74 | (0.26, 1.22) | *0.003* |
| HDL1 Apolipoprotein A2, mg/dL | 6.49 | (2.32, 10.7) | *0.003* | 6.39 | (2.29, 10.5) | *0.003* |
| HDL2 Phospholipids, mg/dL | 2.20 | (0.71, 3.69) | *0.005* | 2.45 | (0.94, 3.96) | *0.002* |
| HDL2 Cholesterol, mg/dL | 3.43 | (0.84, 6.01) | *0.010* | 3.92 | (1.28, 6.56) | *0.004* |
| HDL2 Free Cholesterol, mg/dL | 10.3 | (3.32, 17.3) | *0.005* | 10.8 | (3.88, 17.8) | *0.003* |
| HDL2 Apolipoprotein A2, mg/dL | 5.73 | (0.47, 11.0) | *0.034* | 6.81 | (1.61, 12.0) | *0.011* |
| HDL2 Apolipoprotein A1, mg/dL | 1.91 | (0.65, 3.17) | *0.004* | 2.10 | (0.82, 3.38) | *0.002* |
| HDL3 Phospholipids, mg/dL | 2.10 | (0.59, 3.61) | *0.007* | 2.49 | (0.94, 4.03) | *0.002* |
| HDL3 Cholesterol, mg/dL | 3.16 | (0.71, 5.60) | *0.012* | 3.90 | (1.42, 6.38) | *0.003* |
| HDL3 Free Cholesterol, mg/dL | 9.56 | (1.32, 17.8) | *0.024* | 11.6 | (3.19, 20.0) | *0.008* |
| HDL3 Apolipoprotein A1, mg/dL | 1.71 | (0.47, 2.94) | *0.008* | 2.10 | (0.83, 3.37) | *0.002* |
| VLDL4 Cholesterol, mg/dL | -3.43 | (-6.63, -0.23) | *0.036* | -3.50 | (-6.94, -0.07) | *0.046* |
| VLDL 4 Free Cholesterol, mg/dL | -7.34 | (-14.5, -0.17) | *0.045* | -7.40 | (-15.1, 0.29) | *0.059* |
| Univariable and multivariable linear regression model. Multivariable model adjusted for age, BMI and menopausal status. 95% Confidence Interval. Significance level p<0.05.  Abbreviations: HDL, high-density lipoprotein; VLDL, very-low-density lipoprotein; LDL, low-density lipoprotein; | | | | | | |
